# “Pharmacological” analysis of atrial fibrillation maintenance mechanism: reentry, wavelets, or focal?

**DOI:** 10.3389/fcvm.2025.1447542

**Published:** 2025-01-24

**Authors:** Alexander Burashnikov

**Affiliations:** Lankenau Institute for Medical Research, Wynnewood, PA, United States

**Keywords:** atrial fibrillation, reentry, spiral wave, rotor, focal source, excitable gap, antiarrhythmic agents

## Abstract

The primary electrophysiological mechanism of atrial fibrillation (AF) maintenance is poorly defined. AF mapping studies readily record focal activations (defining them as focal sources or breakthroughs) and “incomplete reentries” (defining them as reentries or would-be-reentries) but do not or rarely detect complete circular activations. Electrophysiological alterations induced by anti-AF drugs before AF cardioversion may help delineate the mechanism of AF maintenance. Cardioversion of AF by antiarrhythmic drugs is associated with prolongation of the AF cycle length and temporal excitable gap (t-EG), resulting in improvement in AF organization (AF-org), and with or without alterations in the refractory period, conduction velocity and wavelength. Such electrophysiological pattern is conceivable with termination of a single focal source but not a single reentry (Class III agents do not increase reentrant t-EG). Yet, a single focal source and multiple focal sources are plausible as the primary mechanism of AF maintenance prior drug administration. Improvement in AF-org caused by anti-AF agents before AF cardioversion is coherent with simultaneous multiple random reentries and wavelets. However, simultaneous multiple reentries are unlikely to occur regularly (most of the contemporary AF mapping studies report either a single reentry at a time or no reentry at all), and the ability of random wavelets to maintain AF is speculative. The conducted analysis inclines toward the focal source as the primary mechanism of AF maintenance.

## Introduction

The primary electrophysiological mechanism sustaining atrial fibrillation (AF) remains unknown (1–3). The dominant role of reentry in the maintenance of AF, thought proven 1–3 decades ago (4–9), is the subject of debate these days (2, 10–12). Contemporary AF mapping studies either rarely or never detect full circular activations and readily record “incomplete reentries”, defining the latter as “reentries” or “would-be-reentries” depending on the chosen definition of reentry (1, 9–22). Practically all AF mapping studies readily detect focal activation patterns that, however, can reflect true focal sources and breakthroughs (1, 9–22). Generally, current AF mapping technologies do not usually permit the precise establishment of the AF mechanism. Electrophysiological alterations induced by antiarrhythmic drugs prior AF cardioversion may help in delineating the primary mechanism of AF maintenance.

### Mechanisms of AF maintenance

Functional reentry was accepted as the primary mechanism of AF maintenance for decades (1, 4, 5, 8, 23–25). From the 1970s to 1990s, the leading circuit reentry was thought to be the main mechanism of AF (4, 5, 23, 24). According to the theory, the leading circuit is determined by the wavelength (WL) and characterized by the absence of the fully excitable gap (EG) and the presence of a refractory core (23, 26). The WL is the product of atrial effective refractory period (ERP) and conduction velocity (CV), which estimates the probability of the leading circuit reentry generation (the shorter the WL, the greater the probability, and vice versa) (26). By about 2000, due to significant inconsistencies of the leading circuit with cardiac fibrillation characteristics, the theory was essentially replaced with the “spiral wave/rotor” concept as the primary mechanism for cardiac fibrillation (1, 25, 27, 28). Spiral wave activity is determined by the sink-source relationship of the inner-curved wavefront tip with the tissue (25, 27, 28). Critical characteristics of the spiral wave are the presence of the EG and an excitable but not excited core (25, 27, 28).

At the beginning of the 20th century, Mines (29), and Lewis (30) suggested that reentry could sustain AF. Around mid-century, Scherf et al. (31) and Prinzmetal et al. (32) reasoned that AF could be maintained by focal source(s). In the 1950s to 1960s, Moe and colleagues proposed that AF was primarily maintained by multiple random wandering wavelets (33, 34). This theory was “upgraded” to the multiple random functional reentry paradigm in the 1980s–1990s (4–7). In fact, it was generally accepted at that point that the main mechanism of AF maintenance was simultaneous random multiple functional reentries (4–7, 24, 35–37). This acceptance seemed largely based on preconceived assumptions rather than reproducible data. In the 1980s–1990s, AF mapping studies detected “incomplete reentries” but not full-circuit reentries (4, 24, 38). After 2000, with improved mapping technologies, reproducible evidence for the existence of simultaneous multiple reentries during AF has not been obtained (10–13, 16, 17, 19–21, 39, 40), suggesting that such multiple reentries are either not detectable with available mapping technologies or do not exist. Recently, Lee et al., using a high-density biatrial epicardial mapping, reported that the maintenance of AF in Moe's original experimental AF model is associated with multiple simultaneous sustained and intermittent focal activation patterns without or only with rare full-circuit reentrant activations (13, 19).

Anatomical reentry may play a critical role in AF maintenance, particular in the setting of significant atrial structural remodeling (40, 41). However, how often anatomical reentries maintain AF is unknown. Most of the AF mapping studies utilizing simultaneous epicardial and endocardial mapping in the setting of significant atrial structural remodeling did not record any anatomical reentries (10, 39, 42, 43). Also, the causative role of atrial structural remodeling in AF generation is controversial (44–48).

In the past 30 years, mostly from the 1990s to early 2010s, some experimental and clinical mapping studies reported that AF could be maintained by a single rotor, causing fibrillatory conduction in the rest of the atria (8, 9, 49). In the last decade, however, the vast majority of AF mapping studies either have recorded sporadic short-lived circular activations (usually 1–3 rotations) or have not recorded any full circular activation at all (11–21), questioning a single stable rotor as the primary mechanism of AF maintenance.

The interpretation of AF mapping data is critical in determining reentry (1, 2, 22, 50–52). Most who consistently detected rotors during AF have used the phase mapping approach for rotor detection (9, 14, 53) [introduced by Gray et al. in the late 1990s (54) and preceded by failure of detecting full-circuit reentries during AF with “conventional” methodologies by this group (38)]. It is well recognized now that the phase mapping method often finds false reentries (12, 18, 55). False reentries (or would-be-reentries) are readily detected during AF (1, 11, 12, 20, 22, 50, 56). In fact, “reentries” are readily detected even at the absence of AF during atrial rapid pacing (51). One would intuitively expect that “incomplete reentries” are natural consequences of any rapid atrial activation(s) in the setting of anatomical and rate-dependent functional heterogeneities in the atrium. How many of these “incomplete reentries” are true reentries is unknown. Interestingly, ablation approaches targeting reentry substrate (i.e., CFAE, FIRM, and fibrosis), initially thought promising, essentially failed to reduce AF occurrence (48, 57–61).

It is well recognized that AF can be maintained by focal sources (11–13, 16, 17, 31, 32, 62–64). Practically all AF mapping studies readily record focal activations (9–13, 15, 16, 20, 21, 39, 62, 63, 65–69) and many AF mapping studies detect either focal activations without reentries or predominantly focal activations (10–13, 16, 17, 20, 66–69). Yet, a lot of the AF mapping investigators record only short-lasting focal activations (commonly ≤3 beats) (10, 15, 21, 39), with most of these focal patterns interpreted as breakthroughs (10, 15, 39). At the same time, many AF mapping studies consistently record both intermittent and sustained focal activations and many of these activations are thought to reflect true focal foci (11–13, 16, 17, 20, 62, 63, 70). Interestingly, electrically isolated conduction pathways, encountered in remodeled atria, may give rise to “trapped reentry” (71). “Trapped reentry” manifests as a focal source (71) that may complicate the discrimination of focal vs. reentrant mechanisms in AF. The contribution of focal sources to the maintenance of AF is poorly understood.

Driven by the absence of evidence for either reentry or focal sources as the primary underlying mechanism of long-standing persistent AF, De Groot et al. have suggested that long-standing persistent AF is maintained by constant aberrant multiwavelets propagating between epicardium and endocardium, secondary to atrial structural remodeling (i.e., the double-layer hypothesis) (10, 39). The basis for the double-layer AF mechanism is a major epi-endocardial electrical dissociation, mainly due to significant atrial structural remodeling. It was estimated that about 400 new wavelets are generated every second during longstanding persistent AF, sufficient to maintain AF (10). Note, while circular activation patterns were not or rarely observed in De Groot et al. studies, focal activation patterns were readily recorded; however, these focal activations were short-lived and the majority were deduced as breakthroughs *(*10, 39, 72). Interestingly, Lee et al. also did not or rarely detect full-circular activation pattens and consistently recorded focal activations during long-standing persistent AF (11, 12, 16). These foci, however, were both sustained (≥32 s) and intermittent (commonly firing simultaneously), and many of these focal activations were deduced as real focal sources maintaining AF (11, 12, 16).

There is some resemblance between the double-layer hypothesis (10, 39) and the original multiple wavelet hypothesis developed by Moe et al. in the 1950s and 1960s (34), i.e., in both theories AF is maintained by the multiple random wandering wavelets. It is practically impossible to prove or disprove the original multiple wavelet and double-layer hypotheses with current AF mapping technologies. Yet, while the ability of random multiple wavelets to maintain AF is speculative (1, 2), random multiple wavelets (or “fibrillatory conduction”) can be readily generated by a single stationary source (8, 13, 19, 49).

There are important limitations of the currently available AF mapping technologies, usually not permitting a precise discrimination of arrhythmic mechanisms maintaining AF (1). These limitations are 1) commonly insufficient resolution, 2) restricted atrial mapping area (usually a part of epicardial surface), 3) the absence or partial information on the intramural activation, 4) uncertainties in the interpretation of signals/electrograms, 5) short duration of uninterrupted mapping time (usually ≤30 s), etc (1, 2). For example, most of the small localized “reentries” detected by “conventional” mapping resolution appear to be pseudo-reentries when viewed in high resolution (22, 50). Yet, failure of detecting a full-circuit reentry or multiple simultaneous reentries does not exclude their presence in unmapped areas, particularly intramurally. Another example is that although “clean” focal activations on atrial surface are readily detected during AF with a high resolution, these focal activations can be breakthroughs from a remote source(s) (10). The presence or absence of focal and reentrant activations during AF must be confirmed with comprehensive and reliable 3-dimentional activation maps, not achievable currently (1, 2).

The autonomic nervous system (ANS) may importantly contribute to the generation of AF (most of the supporting data is related to the initiation of AF, with little data being related to the maintenance of AF) (73, 74). Pertinent data on the involvement of ANS in AF maintenance that may help distinguish reentry vs. focal source maintaining AF are scarce, indirect, and doubtful. For example, acetylcholine promotes AF maintenance by shortening atrial repolarization and hyperpolarizing atrial resting membrane potential (38, 49, 75), suggesting reentry but not focal sources as the underlying mechanism of AF maintainance (38, 76). However, during cholinergically-mediated AF mapping studies commonly either do not record complete reentry at all or observe it rarely while readily recording focal activation patterns (12, 13, 38, 77). The issue of AF cardioversion with I_KACh_ blockers is controversial, i.e., I_KACh_ blockers may or may not stop AF (78–81), and the specificity of I_KACh_ blockers is a concern [e.g., XAF-1407 prolongs QRS (79), indicating a major block of the sodium channel]. While *β*-blockers are commonly inefficient in cardioversion of AF, the interpretation of these data in term of mechanisms maintaining AF is unclear. Sympathetic nervous system may or may not affect both focal and reentrant mechanisms (this issue is poorly understood) (73, 82, 83).

Thus, while reentry was considered proven as the primary mechanism of AF maintenance from at least the 1970s to the 2010s (4–9), its dominance now is debatable (2, 10–12). Contemporary AF mapping studies readily detect focal activations and “partial reentries” but not full circuit activations (10–13, 16, 17, 19, 20, 66, 68, 84). With available technologies, it is difficult or impossible to definitively prove reentrant, wavelet, and focal mechanisms of AF maintenance (1, 2). The primary mechanism of AF maintenance remains poorly defined.

### Drug-induced atrial electrophysiological alterations before AF termination

Atrial electrophysiological alterations induced by antiarrhythmic drugs before AF termination may shed light on electrophysiological mechanisms of AF maintenance. It is important to identify changes in electrophysiological parameters that consistently precede drug-induced cardioversion of AF as well as the value of these parameters for the differentiation of AF arrhythmic mechanisms. Alterations of the principal electrophysiological parameters related to AF (such as ERP) in the very last, or last several, AF beats before AF termination are practically unknown (e.g., ERP cannot be determined in the several last beats of AF) or inadequately defined [one study reported CV acceleration immediately before AF cardioversion (85)]. Therefore, the current analysis largely examines sustained drug-induced alterations of the electrophysiological parameters during AF maintenance before AF cardioversion [such alterations have been reported (85–95) or can be reasonably deducted, as discussed later]. To reduce uncertainties in interpretation, mostly data with Class I and III agents were analyzed.

### Effective refractory period

Prolongation of atrial ERP by anti-AF agents is a surrogate for estimating anti-AF efficacy of the drugs (3, 96). I_Kr_ blockers increase ERP by prolongation of the action potential duration (APD) and I_Na_ blockers by induction of the post-repolarization refractoriness (97). When achievable, a significant drug-induced atrial ERP prolongation is likely to be associated with AF cardioversion or the AF rate slowing. However, prolongation of atrial ERP/APD may or may not be attainable with Class I and III agents during AF (86, 98, 99), and both classes can terminate AF without prolongation of atrial ERP (86, 100–102). Interestingly, I_Kr_ blockers are generally more efficient in cardioversion of persistent AF than I_Na_ blockers (86, 99, 103, 104), despite that Class III should not and Class I should significantly prolong atrial ERP at very rapid activation rates [due to their reverse and use dependency, respectively (3, 105, 106)]. Thus, atrial ERP prolongation cannot be considered a prerequisite for AF termination by anti-AF agents. Also, because atrial ERP prolongation acts to terminate, or at least slow down, all arrhythmic mechanisms, drug-induced ERP prolongation does not seem useful for discriminating among reentrant, wavelet, and focal mechanisms.

### Conduction velocity

**Figure 1:**
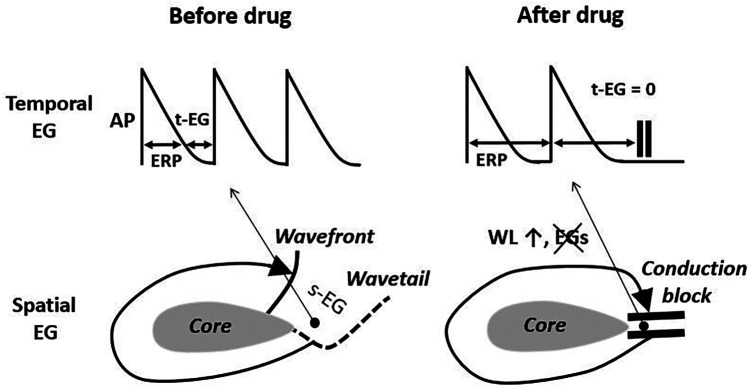
The elimination of the temporal and spatial excitable gaps (t-EG and s-EG, respectively) leading to conduction block is a well recognized mechanism of drug-induced reentry termination. The basis for this mechanism is wavelength (WL) prolongation, secondary to the effective refractory period (ERP) increase. AP, action potential. Pease see text for details.

**Figure 2:**
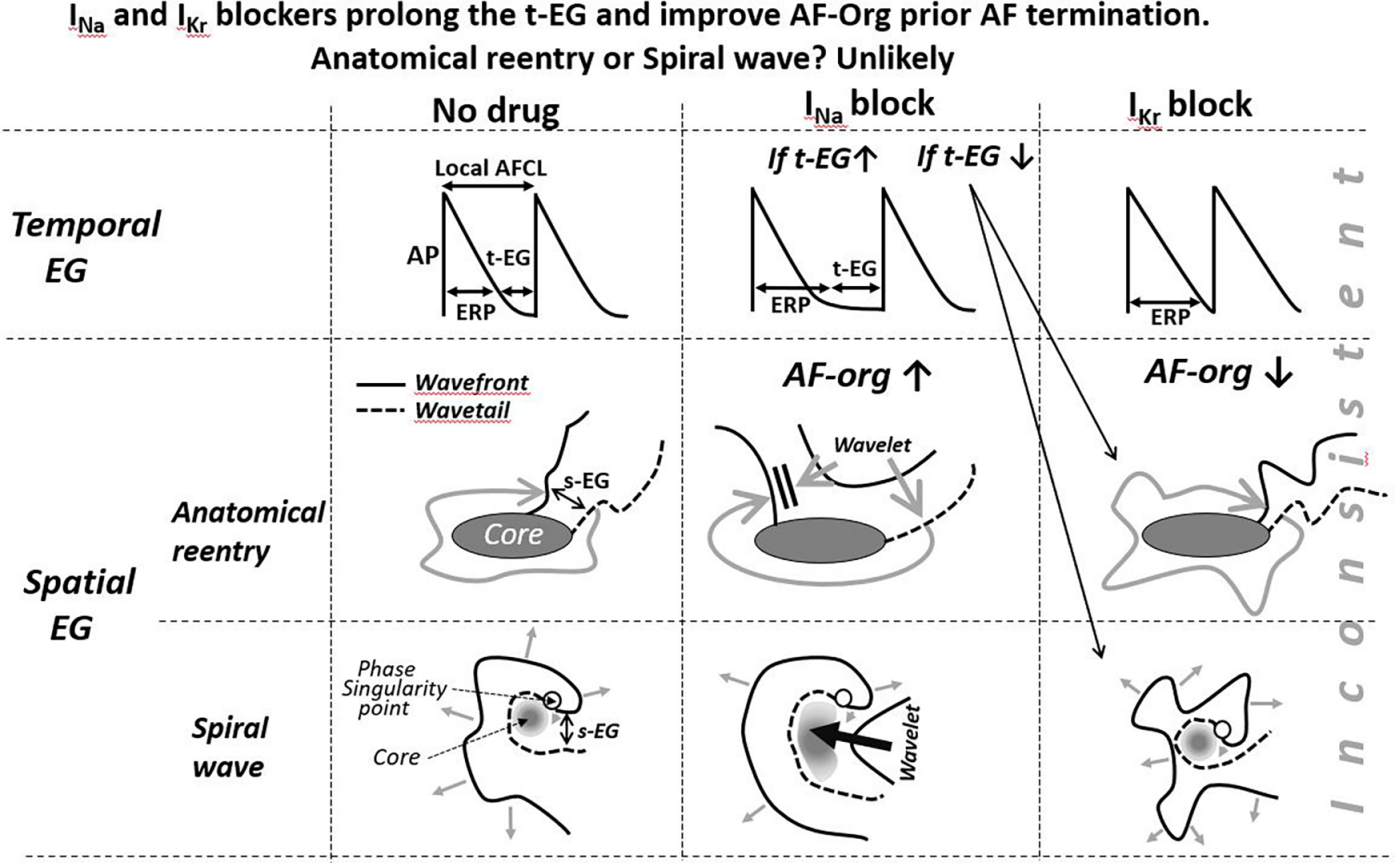
Schematic simplified illustrations of drug-induced alterations of the t-EG, s-EG, and AFCL in anatomical and spiral wave reentry. Maintenance and termination of reentries with prolongation of the EGs are not feasible with I_Kr_ blockers (I_Kr_ blockers decrease the EGs) and controversially conceivable with I_Na_ blockers. To be consistent with mother reentry being the main mechanism of AF, a reasonable explanation(s) for AF maintenance and termination associated with prolongation of the t-EG should be for both I_Na_ and I_Kr_ blockers. Pease see text for details.

Slowing of atrial CV during AF generally occurs with inhibition of I_Na,_ but not with specific block of I_Kr_ (86). Whether I_Na_ block-induced CV slowing is involved in AF termination is not clear. In the case of reentry, I_Na_ block-induced CV slowing during AF may increase the chance of conduction block (that may lead to reentry termination; Figure 1), but slowing CV itself should promote reentry generation (a basic postulate for reentry) (107). Also, I_Na_ block-induced prolongation of the temporal excitable gap (t-EG) during AF (Figure 2; as discussed later) should reduce the chance of conduction block (Figure 1). Interestingly, CV consistently accelerates just before AF cardioversion with potassium and sodium channel blockers (85), likely due to a concomitant prolongation of the t-EG, as discussed later. In the case of a focal source, CV alterations by itself should not directly affect rapid focal source firing. Thus, the limited knowledge about the involvement of CV in drug-induced AF cardioversion is insufficient for the differentiation of arrhythmic mechanisms maintaining AF.

### Wavelength

In the 1980s and 1990s, wavelength (WL) was generally considered a predictable parameter for anti-AF efficacy of antiarrhythmic drugs based on numerous studies reporting that both I_Na_ and I_Kr_ blockers terminated and prevented AF by prolongation of the WL (Figure 1) (5, 108–110). It has become evident over the last two decades that anti-AF agents may effectively terminate AF with prolongation and shortening of the WL as well, without a change of the WL (3, 86, 100). Therefore, WL is not a reliable parameter for predicting drug-induced AF cardioversion. Note that drug-induced prolongation of WL is caused exclusively by ERP lengthening (CV slowing shortens the WL) (3, 5, 109). So, if drugs prolong WL, it is also invariably associated with ERP lengthening that alone may account for the termination of any arrhythmic mechanisms.

### AF cycle length

Cardioversion of AF with I_Na_ and I_Kr_ blockers is quite consistently accompanied by prolongation of AF cycle length (AFCL) (85, 86, 89, 92, 94, 111–116). Yet, drug-induced prolongation of AFCL (i.e., slowing of AF activity) before AF termination is expected with any arrhythmic mechanisms, with apparent exception of anatomical reentry termination by I_Kr_ blockers (117). Therefore, drug-induced prolongation of AFCL by itself does not seem to be a reliable parameter for the differentiation of arrhythmic mechanisms.

### The excitable gap and AF organization

There are temporal and spatial excitable gaps during AF (t-EG and s-EG, respectively) (86). The t-EG is defined as the difference between the AFCL and ERP at any given point, and the s-EG is defined as the excitable spatial distance between wavefront and wavetail at any given area (i.e., AFCL x CV minus ERP x CV). Spatiotemporal t-EG and s-EG during AF can be estimated but not measured nowadays (it is impossible to measure ERP simultaneously in multiple locations during AF, among many reasons).

Available data indicate that cardioversion of AF with antiarrhythmic agents is preceded by a sustained general prolongation of the t-EG (86, 88, 90) and improvement in AF organization (AF-org) (85–95, 113). The latter by itself demonstrates a sustained general prolongation of the t-EG. Indeed, improvement in AF-org is secondary to the prolongation of a general spatiotemporal t-EG that provides a longer recovery time for the activating wavefronts to become more organized. Drug-induced alteration of the s-EG prior AF termination is more difficult to estimate than the t-EG. Although drug-mediated improvement in AF-org is likely to be associated with a general increase in the s-EG, it is time (i.e., t-EG) and not space (i.e., s-EG) that determines atrial electrical recovery. According to the estimation of Wijffels et al., the s-EG before AF cardioversion (within minutes) was statistically significantly widened by d-sotalol, cibenzoline, and hydroquinidine, but not flecainide (86).

Directly comparing the effect of flecainide, d-sotalol, cibenzoline, and hydroquinidine on principal atrial electrophysiological parameters (ERP, WL, CV, s-EG, and t-EG) before termination of AF (within minutes) in goats, Wijffels et al. found that t-EG prolongation was the only parameter consistently associated with AF cardioversion (86). van Hunnik et al. (85) studied changes in AF organization, AFCL, and CV occurring immediately (within seconds) before spontaneous and drug (potassium and sodium channel blockers)-induced AF termination in goats. AF organization was consistently improved immediately before AF termination in all studied settings, coinciding with AFCL increase and CV acceleration (85). The improvement in AF organization and acceleration in CV were likely due to prolongation of a general t-EG.

**Figure 3:**
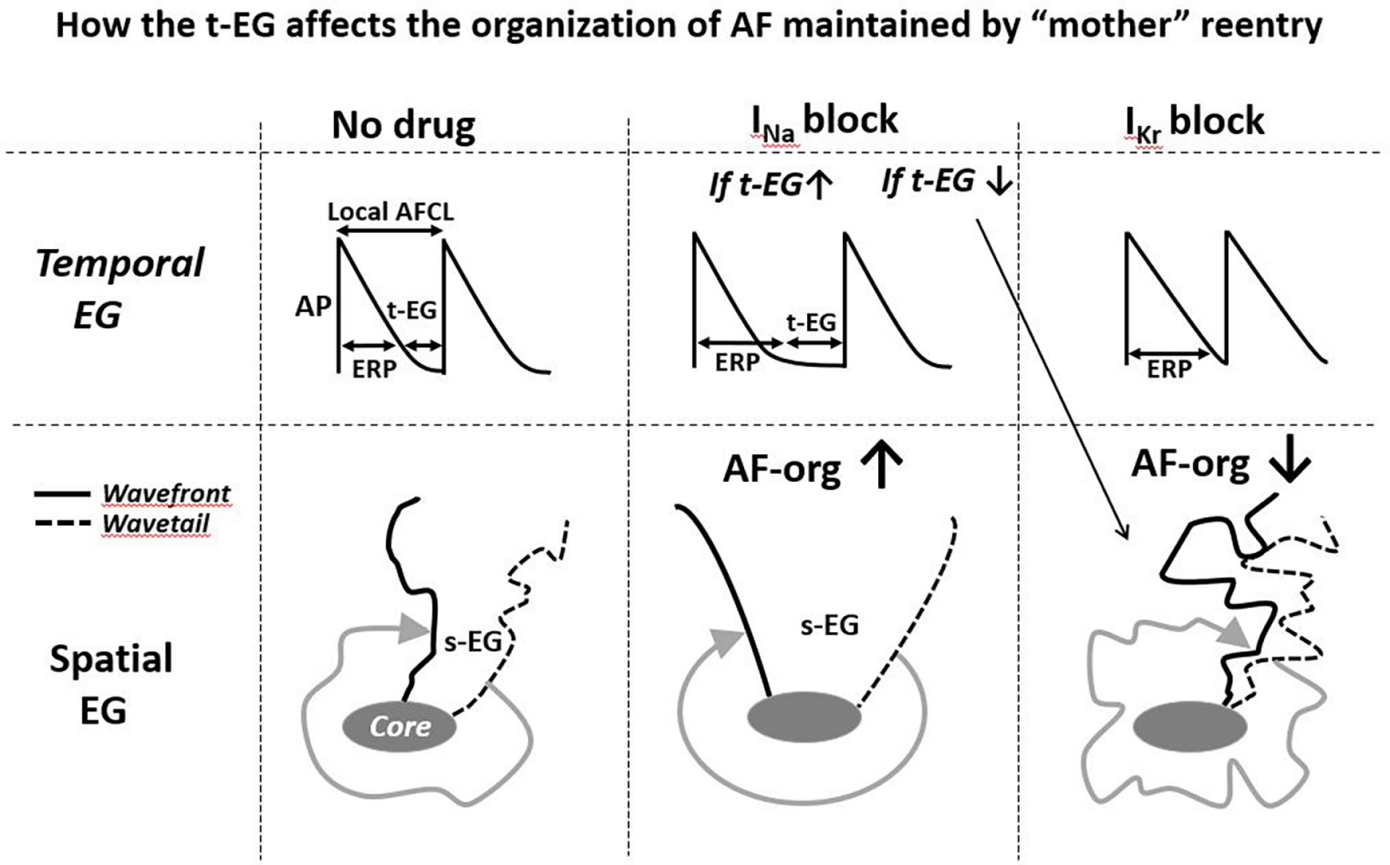
Simplified illustrations of how alterations in the t-EG affect the organization of AF (AF-org) sustianed by mother reentry. Prolongation of the t-EG improves and shortening of the t-EG aggravates AF-org (prolongation of the t-EG should be commonly associated with inrease of the s-EG). I_Na_ blockers may both increase and decrease the reentrant EGs. I_Kr_ blockers decrease the reentrant EGs. Please see text for details.

In the case of AF sustained by a mother reentry, both the t-EG and s-EG around the reentry core are vital parts of the circuit (Figures 1–3). Drugs may decrease, increase, and not change the EGs around the reentry core (Figures 1–3) (25, 107, 117–119). The elimination of the t-EGs at any point around the reentry core (the t-EG cannot disappear without elimination of the s-EG and vice versa) should lead to conduction block at this point, which may result in termination of the circuit (Figure 1). Prolongation of a general t-EG along the reentrant pathway may make the circuit more stable (by reducing the chance of conduction block; Figure 1) or more vulnerable to termination. The latter may occur secondary to destabilization of the reentrant core by invading wandering wavelets (relevant to functional reentries; Figure 2) (120) or due to collision of the reentrant wavefront with some antidromic wavelets (relevant to anatomical and functional reentries; Figure 2) (119). Importantly, it is drug-induced termination of reentry with elimination of the EGs along the reentrant core that is well-recognized (Figure 1) (96, 107, 109) and drug-induced termination of reentry with prolongation of the EGs around the reentrant core is poorly defined and controversial (Figure 2; as discussed later).

**Figure 4:**
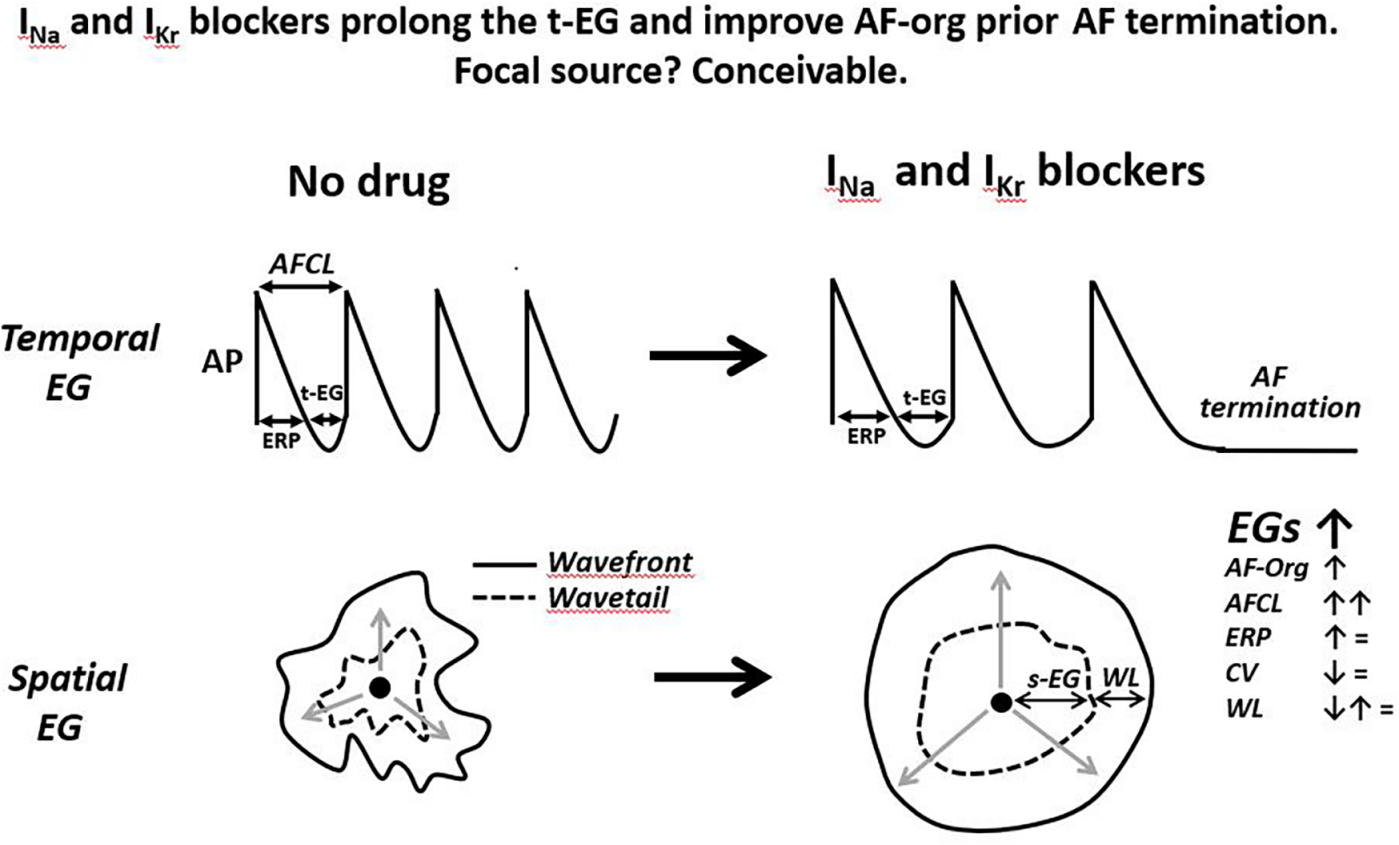
Schematic simplified illustrations of drug-induced alterations of the t-EG, s-EG, AF-org, and AFCL prior the termination of a single focal source firing. Anti-AF drugs prolong AFCL and t-EG, improving AF-org in AF maintained by a focal source. The slowing of rapid focal source firing is a plausible sign of its subsequent cessation. Drug-induced improvement in AF-org and termination of focal AF may or may not be associated with changes in atrial ERP, CV, and WL. The illustrated action potentials (AP) are from the focal source location, displaying diastolic depolarization. Please see text for details.

In a mother focal source sustaining AF the t-EG is an integral part of this source, affecting the rate of firing (by modulating the recovery of the ion channel currents involved in the firing) and being affected by the rate of firing (i.e., the slower the rate, the greater the t-EG, and vice versa). How and if the s-EGs surrounding a rapid focal source affect the generation of this focal source firing is unknown. Yet, source-sink relationship between focal source and surrounding tissue may change the s-EGs. It has been shown that autonomic influences and frequency of activation may alter source-sink relationship between focal source and surrounding tissue (121, 122), potentially changing the t-EG*.* How and if anti-AF drugs modify the s-EG surrounding focal source is unknown. Available data indicate that anti-AF agents reduce the firing rate of arrhythmic focal activity (123–126) that should prolong the t-EG in the spot of a rapid focal source (Figure 4)*.* Cessation of rapid focal activity is commonly associated with its deceleration (Figure 4) (127–131).

If AF is sustained by multiple simultaneous sources (reentrant, focal, or wavelet), prolongation of the spatiotemporal t-EG (i.e., improvement in AF-org) is likely to be associated with the reduction in the number of these sources, promoting the cessation of such AF. So, a general prolongation of the t-EG before AF termination appears to be consistent with all multisource AF mechanisms.

Thus, antiarrhythmic agents consistently induce prolongation of the t-EG and improvement in AF-org prior AF termination, and these parameters may help differentiate arrhythmic mechanisms.

### Drug-induced prolongation of the t-EG and improvement in AF-org before AF termination: reentry, wavelets, or focal?

The following part of the analysis is an attempt to estimate how the drug-induced prolongation of the t-EG and improvement in AF-Org before AF termination correspond to reentrant, wavelet**,** and focal mechanisms.

### A single anatomical reentry? unlikely

The classical mechanism of anatomical reentry termination by drugs is conduction block caused by the elimination of EGs in a critical area (secondary to prolongation of the WL; Figure 1) (107, 117, 118). This mechanism is inconsistent with prolongation of the t-EG induced by drugs prior AF cardioversion. With a general t-EG prolongation along the anatomical circuit, the probability of conduction block and, thus, termination of the reentry by this mechanism ought to be reduced (Figures 1, 2).

Class III agents shorten, not prolong, the t-EG in anatomical reentry [Figure 2; as it is logically conceivable and has been reported in atrial flutter studies (99, 117, 118)]. Therefore, lengthening of the t-EG with I_Kr_ blockers around the anatomical reentrant circuit is contradictory by itself. Class I agents may not only shorten and but also prolong the t-EG around the anatomical reentrant core (Figure 2) (132). In the case of such t-EG prolongation, anatomical reentry can be terminated by collision of the reentrant wavefront with antidromically propagating activation of any origin (Figure 2) (132). However, entrainment should readily occur also (Figure 2). To be coherent with a mother anatomical reentry, a reasonable explanation for its termination associated with the prolongation of the t-EG should be for both I_Na_ and I_Kr_ blockers. Considering this argument as well as the inconsistency with the classical mechanism of drug-induced termination of anatomical reentry with prolongation of the t-EG, anatomical reentry is unlikely as the primary mechanism of AF maintenance.

### A mother functional reentry? Unlikely

There is little to no data and thoughts on termination of functional reentries by drugs associated with prolongation of the t-EG. In contrast, there are plenty of data and theories on termination of functional reentry by Class I and III agents associated with elimination of the t-EG, resulting to conduction block (secondary to prolongation of the WL; Figure 1) (107, 109, 133). This mechanism was directly or indirectly supported by many studies, largely in the 1980s and 1990s (96, 107, 109, 133). During that time, the primary interpretation of the functional reentry was based on the leading circuit reentry theory, and drug-induced termination of functional reentry due to elimination of the EGs leading to conduction block was consistent with this theory (Figure 1). By about 2000, due to significant inconsistencies of the leading circuit with cardiac fibrillation characteristics, the theory was essentially replaced with the “spiral wave/rotor” concept as the primary mechanism for functional reentry (1, 25, 27, 28). To what extent drug-induced termination of functional reentry via conduction block (Figure 1) is applicable to spiral waves is unclear.

The termination of mother rotor by anti-AF agents is poorly defined, perhaps due to the absence or rare detection of a sustained spiral wave during AF (10–13, 15–22, 38, 77, 94, 134, 135). All available data and theories suggest that I_Kr_ block (by prolonging ERP with no or little CV slowing) shortens the t-EG in spiral waves, as in all other reentrant mechanisms (25, 107, 117, 132, 136), that is inconsistent with t-EG prolongation induced by I_Kr_ blockers before AF termination (86). In the original study demonstrating that d-sotalol-induced termination of AF was associated with prolongation of the t-EG and s-EG, the only explanation for these EG prolongations, reconciling it with reentry, was that d-sotalol might inhibit I_Na_ (86) (high concentrations of d-sotalol block I_Na_ (137). However, d-sotalol did not affect atrial CV in this study (86), indicating that d-sotalol caused little to no inhibition of I_Na_.

Experimental data indicate that I_Na_ blockers can shorten (109, 138, 139), prolong (120), or leave EGs unchanged (138) in functional reentrant circuits. There was one experimental study reporting that termination of vagally-mediated AF by pilsicainide (an I_Na_ blocker) was associated with prolongation of the t-EG and reduction of the WL (120). In this study, a mother rotor-based AF stopped when the spiral wave core was excited by a wavelet invader coming from widened spatiotemporal EGs (120) (as schematically illustrated in Figure 2). However, these results contradict to another study reporting that pilsicainide shortened the t-EG and prolonged the WL before cardioversion of vagally mediated AF (140).

I_Kr_ block-induced improvement of AF-org (86, 90, 95, 141) are discordant with the effects of I_Kr_ blockers to shorten the EGs of a mother spiral wave (Figures 2, 3) (25, 136). I_Na_ blockers may improve the AF-org of AF maintained by a mother rotor if I_Na_ blockers prolong a general t-EG and the degree of this prolongation is great enough to overwhelm the effect of I_Na_ blockers to worsen AF organization (Figure 3).

An apparent problem with the idea of mother spiral wave termination by antiarrhythmic drugs for the primary explanation of AF termination is the issue of sustainability of spiral waves. Spiral waves are generally unstable by nature (1, 27, 28), and the vast majority of AF mapping studies never recorded sustained rotors (i.e., rotors, when detected, commonly lasted for ≤3 rotations) (11, 15, 17, 18, 20, 21, 66, 94, 134, 135, 142, 143).

It is well recognized that the ERP, CV, WL, and EGs may vary significantly in space and time along function reentry pathway (25, 144). Yet, a spatiotemporal measurement of these parameters around the functional reentry core is technically unrealistic currently [putting aside that any chance of measuring these parameters is very low; the vast majority AF mapping studies never detect a sustained spiral wave (10–13, 15–22, 38, 77, 94, 134, 135)]. When selecting and manipulating the functional reentry-relevant parameters/conditions in mathematical models, there may be some explanations for drug-induced termination of reentry with the t-EG prolongation that are not considered in this review. Such theoretical explanations should reasonably substantiate the termination of functional reentry by both the I_Na_ and I_Kr_ blockers as well as in common and not specific conditions. Particularly, one should explain how I_Kr_ blockers prolong the t-EG in mother reentry-maintained AF.

Thus, drug-induced prolongation of the t-EG before AF cardioversion cannot be reasonably explained with the classical mechanism of drug-induced termination of functional reentry (Figure 1). Also, the termination of mother functional reentry with prolongation of the t-EG is not plausible with Class III blockers and only controversially conceivable with I_Na_ blockers. Therefore, mother functional reentry is unlikely to be the primary mechanism of AF maintenance.

### Simultaneous random multiple reentries and wavelets? Speculative

Anti-AF drug-induced improvement in AF-org should reduce the appearance of random short-living reentries and, thus, is consistent with the termination of AF maintained by such reentries. However, contemporary AF mapping studies do not record simultaneous multiple reentries *(*10–13, 16, 17, 19, 39, 40). Moreover*,* increasingly more AF mapping studies either do not observe full circular activity at all or observe only a single rotational activity at a time (10–13, 16, 17, 19, 39, 40), with many or all of these rotational activities being would-be-reentries (12, 13, 19).

Drug-induced improvement in AF-org before AF cardioversion is also generally consistent with the random multiple wavelet theories (Moe's and double layer). The original Moe's hypothesis was largely abandoned in the 1980s to 1990s (in favor or multiple random reentries) (4–7, 24, 35–37), but still remains in circulation (134). The double-layer hypothesis seems limited, or largely limited, to long-standing persistent AF (10). It is impossible to definitively prove or disprove the multiple wavelet hypothesis(es) with available mapping technologies (1, 2). At the same time, random multiple wavelets (i.e., fibrillatory conduction) can be generated by a rapid stationary source (13, 19, 49), including by rapid pacing (51).

There are additional issues with random multiple reentries and wavelets as the primary mechanisms of AF maintenance. First, there is substantial evidence for the usual presence of repetitive activation patterns and some spatiotemporal stability during paroxysmal and persistent AF (54, 145–154), arguing against a completely random nature of the primary mechanism of AF maintenance. Second, distinct and repetitive F waves displaying a narrow single or dominant power peak are usually recorded during paroxysmal and persistent AF in most patients (95, 111, 155–159) that does not appear to be consistent with random multiple reentries and wavelets. Third, if AF is sustained by simultaneous random multiple sources, the termination of such AF should be due to the cessation of the last random driver and not by a stable driver. However, available data indicate that anti-AF drugs by significantly slowing the rate of AF (86, 94, 100, 116, 159–161) commonly convert AF to a tachycardia-like arrhythmia before the arrhythmia termination (100, 113, 116, 125, 162, 163). Such tachycardia-like arrhythmias are likely to be maintained by a single stationary driver. All these data and arguments cast doubt on the primary role of random multiple reentries and wavelets in AF maintenance.

### Focal source(s)? Conceivable

Drug-induced prolongation of the t-EG resulting to improvement in AF-org before AF termination, with or without changes in the ERP, CV, and WL, is conceivable with termination of a focal source (Figure 4). It is known that drug-induced prolongation of the t-EG is inevitably associated with increase of the AFCL (t-EG = AFCL-ERP while anti-AF drugs do not shorten ERP) and that anti-AF agents prolong AFCL before AF termination (86, 89, 92, 94, 111–116, 164). These facts can be explained with focal sources (Figure 4). Indeed, available data indicate that termination of arrhythmic focal source activity with or without drugs is preceded by slowing this activity (123–131) that should be associated with prolongation of the t-EG (Figure 4). Increase in the cycle length and t-EG in the location of focal source maintaining AF should spread throughout the atria (with spatiotemporal variations), leading to a general improvement in AF-org (Figure 4).

We know little about the mechanism(s) of rapid focal sources and mechanisms by which anti-AF drugs can slow down such focal activity. Mechanistically, drug-induced ERP prolongation, when it occurs, should slow down rapid focal source activity, promoting the termination of such activity. Abnormalities in intracellular calcium activity (Ca_i_) and *“*funny” current (I_f_) may be involved in the maintenance of AF (165–167) and many anti-AF agents are capable of reducing Ca_i_ (such as flecainide, ranolazine, amiodarone, etc.) (168, 169) and inhibiting I_f_ (such as amiodarone, flecainide, ibutilide, etc.) (170, 171).

Specific data and theories on focal AF termination by anti-AF drugs are scarce. AF termination by drugs are explained almost exclusively based on the reentry ideology (25, 96, 107, 109), even when the drugs do not prolong atrial ERP before AF termination (86). The investigation of pharmacological cardioversion of focal AF is problematic. In the whole atrium (*in situ*, *in vivo*, or *in vitro*), focal AF is commonly difficult to prove definitively (as discussed previously), and in isolated thin atrial slices or single cells (in which focal sources can be convincingly proved), sustained rapid focal activity with a rate equivalent to AF does not develop (127, 129, 172–174). In fact, focal arrhythmic activity in isolated thin atrial tissue slices and single cells is commonly induced artificially (i.e., by rapid pacing in the presence of a high concentration of *β*-receptor agonists), its rate of activation is typically slower than the rate of atrial tachycardia, and the duration of this focal activity is usually only in seconds (127, 129, 173). These data may argue against focal sources as important drive(s) for AF. However, the fact that focal activity from atrial tissue slices and single cells cannot account even for the rate and sustainability of focal atrial tachycardias refute this assumption. There are focal atrial tachycardias (131) and there is reasonable evidence for the existence of focal AF (11–13, 16, 17, 62–64). It appears that atrial arrhythmic focal sources are capable of generating sustained rapid activation only in natural or close to natural environments (i.e., *in vivo*, *in situ*, or in coronary-perfused atrial preparations) but not in superfused atrial tissue or isolated single cells (3).

**Figure 5:**
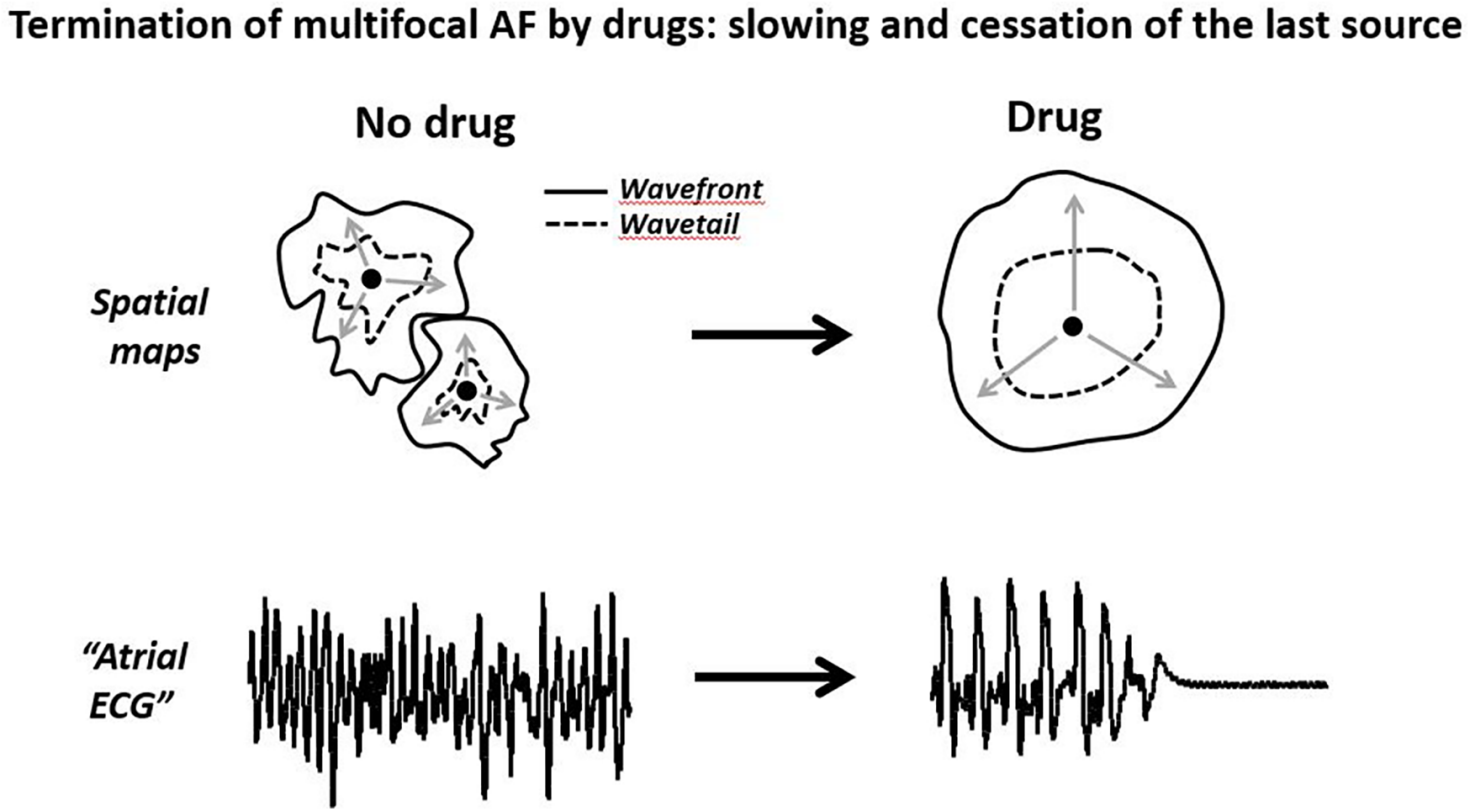
Schematic illustrations of cardioversion of a multifocal AF by anti-arrhythmic agents. The termination of multisource AF is likely due to the slowing and cessation of the last source of the same mechanism maintaining AF. Please see text for details.

Thus, available data indicate that the termination of atrial arrhythmic focal activity by drugs is preceded by the prolongation of the cycle length that should be naturally associated with the lengthening of the t-EG and improvement in AF-org (Figure 4). Also, drug-induced cardioversion of focal AF is plausible with or without alteration in ERP, CV, and WL. Therefore, the reported behaviors of atrial electrophysiological parameters prior AF termination in the presence of Class I and III agents are conceivable with a single focal source (Figure 4). Is this deduction pertinent to the mechanism of AF maintenance before drug application? The primary mechanism of AF maintenance could be a mother source before and after the drug application, with slower activation rate following the treatment. It also could be simultaneous multiple sources before the drug treatment, converting to slower activating fewer sources or a single source following the drug application. If AF is maintained by simultaneous multiple sources, drug-induced termination of AF is likely due to the slowing and cessation of the last source of the same mechanism that maintains AF before drug application (Figures 4, 5). Therefore, a mother focal source and multiple focal sources are conceivable as the primary mechanism of AF maintenance before drug application (Figure 5).

## Conclusions

(1) Drug-induced prolongation of the t-EG and improvement in AF-org prior AF termination, with or without changes in the ERP, CV, and WL, are consistent with a mother focal source and inconsistent with a mother reentry as the main mechanism of AF maintenance. Yet, a single and multiple focal sources are conceivable as the primary mechanism of AF maintenance prior drug administration.
(2) Drug-induced improvement in AF-org during AF maintenance before AF cardioversion is also coherent with simultaneous random multiple reentries and wavelets. However, AF mapping data indicate that simultaneous multiple reentries are unlikely to occur regularly, and the ability of random multiple wavelets to maintain AF is uncertain.
(3) The conducted “pharmacological” analysis in conjunction with AF mapping data supports the notion that focal sources are more likely to be the primary mechanism of AF maintenance than reentries.
